# A structurally distinct TGF-β mimic from an intestinal helminth parasite potently induces regulatory T cells

**DOI:** 10.1038/s41467-017-01886-6

**Published:** 2017-11-23

**Authors:** Chris J. C. Johnston, Danielle J. Smyth, Ravindra B. Kodali, Madeleine P. J. White, Yvonne Harcus, Kara J. Filbey, James P. Hewitson, Cynthia S. Hinck, Alasdair Ivens, Andrea M. Kemter, Anna O. Kildemoes, Thierry Le Bihan, Dinesh C. Soares, Stephen M. Anderton, Thomas Brenn, Stephen J. Wigmore, Hannah V. Woodcock, Rachel C. Chambers, Andrew P. Hinck, Henry J. McSorley, Rick M. Maizels

**Affiliations:** 1 Institute of Infection and Immunology Research and Centre for Immunity, Infection and Evolution, School of Biological Sciences, University of Edinburgh, Edinburgh, EH9 3JT UK; 2 Department of Clinical Surgery, University of Edinburgh, Edinburgh, EH16 4TJ UK; 3 Wellcome Centre for Molecular Parasitology, Institute of Infection, Immunity and Inflammation, University of Glasgow, Glasgow, G12 8TA UK; 4 Department of Structural Biology, University of Pittsburgh School of Medicine, 3501 Fifth Aveue, Pittsburgh, PA 15260 USA; 5 MRC Centre for Inflammation Research, Queen’s Medical Research Institute, University of Edinburgh, Edinburgh, EH16 4TJ UK; 6 Department of Pathology, Western General Hospital, University of Edinburgh, Edinburgh, EH16 4TJ UK; 7 Centre for Inflammation and Tissue Repair, UCL Respiratory, Rayne Institute, University College London, London, WC1E 6JF UK; 8 Present Address: Malaghan Institute of Medical Research, PO Box 7080, Wellington, 6242 New Zealand; 9 Present Address: Department of Biology, University of York, York, YO10 5DD UK; 10 Present Address: Section for Parasitology and Aquatic Diseases, Department of Veterinary Disease Biology, University of Copenhagen, Copenhagen, DK-1870 Denmark

## Abstract

Helminth parasites defy immune exclusion through sophisticated evasion mechanisms, including activation of host immunosuppressive regulatory T (Treg) cells. The mouse parasite *Heligmosomoides polygyrus* can expand the host Treg population by secreting products that activate TGF-β signalling, but the identity of the active molecule is unknown. Here we identify an *H. polygyrus* TGF-β mimic (*Hp-*TGM) that replicates the biological and functional properties of TGF-β, including binding to mammalian TGF-β receptors and inducing mouse and human Foxp3^+^ Treg cells. *Hp-*TGM has no homology with mammalian TGF-β or other members of the TGF-β family, but is a member of the complement control protein superfamily. Thus, our data indicate that through convergent evolution, the parasite has acquired a protein with cytokine-like function that is able to exploit an endogenous pathway of immunoregulation in the host.

## Abstract (web summary)

*Heligmosomoides polygyrus* can activate mammalian TGF-β signalling pathways, but how it does so is not known. Here the authors identify and isolate a *H. polygyrus* TFG-β mimic that can bind both mammalian TGF-β receptor subunits, activate Smad signalling and generate inducible regulatory T cells.

## Introduction

Parasitic helminths are able to establish a state of immune hypo-responsiveness or tolerance in their host to attenuate both host immunity and reactivity to third-party specificities, such as allergens and autoantigens^1–3^. A wide range of molecular and cellular mechanisms of parasite immune suppression have been described, but a prominent feature of many helminth infections is expansion of the regulatory T (Treg) cell population, an immune subset that controls immunity in infection, allergy, and autoimmunity^4–6^. Activation of Treg cells is particularly marked in mice infected with the gastrointestinal nematode *Heligmosomoides polygyrus*, with Treg cells controlling both susceptibility to infection and propensity to allergic reactivity^7–10^. In particular, antibody-mediated depletion of Treg cells promotes resistance to infection in genetically susceptible mice, and Treg population expansion (with IL-2:anti-IL-2 complex) renders genetically resistant mice susceptible^10^.

In the mature peripheral immune system, induction of Treg cells to exogenous antigen specificities, for example from the microbiota or innocuous environmental substances, is promoted by the cytokine TGF-β^11–14^. Mice deficient in either the TGF-β1 ligand or TGF-β receptors have few inducible Treg cells and succumb to disseminated inflammatory disease in the weeks following birth^15^. TGF-β is a member of a highly diversified signaling family, which includes many essential developmental and morphogenetic proteins, and indeed mice lacking the TGF-β2 and TGF-β3 isoforms develop lethal congenital deformities^16, 17^. TGF-β family members have developmental functions in invertebrates, including helminths such as *Caenorhabditis elegans* ^18^ and *H. polygyrus* ^19^, indicating that the immunological function of mammalian TGF-β emerged at a relatively recent point in evolution^20^.

We previously reported that *H. polygyrus* releases a soluble secreted product which activates the mammalian TGFβ receptor pathway to induce expression of the Treg-specific transcription factor, Foxp3, in naive peripheral T cells^9^; however, the identity of the active protein among more than 370 different products released by the parasite^21, 22^ is unclear.

Here we identify and characterize a secreted functionally active 404-aa protein, which although highly cysteine-rich, has no sequence similarity to mammalian TGF-β and does not contain a cystine knot, like all other members of the highly diversified TGF-β family. However, the newly identified product is a fully functional mimic of the mammalian cytokine, and is able, in a parallel fashion, to bind the TGF-β receptors and activate signalling; as a result the mimic we call *H. polygyrus* TGF-β mimic (*Hp-*TGM) potently induces expression of Foxp3 in mouse and human T cells. This finding indicates convergent evolution of immunosuppressive mediators by host and parasite, generating a distinct TGF-β mimic that may have therapeutic value in inhibiting inflammatory disorders in humans.

## Results

### Identification of a TGF-β mimic from *H. polygyrus*

**Fig. 1:**
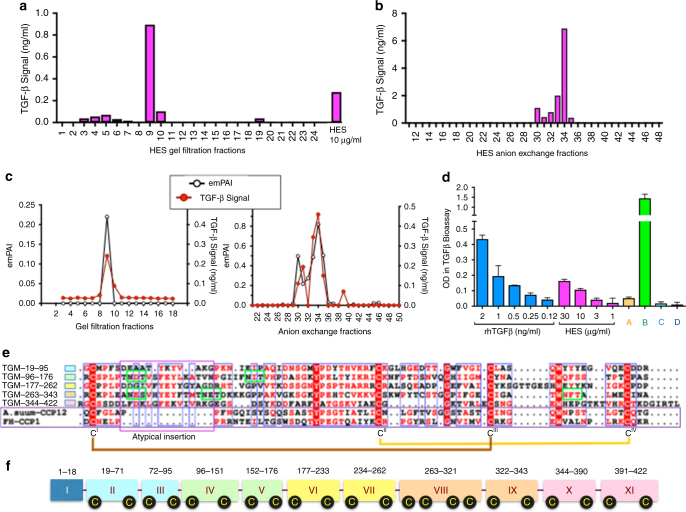
Identification of *Hp*-TGM. **a** Fractionation of HES by gel filtration FPLC. 1 mg of HES was separated on a Superdex 200 10/300 GL column and 1 ml fractions collected for assay with MFB-F11 reporter cells; responses were calibrated with recombinant human TGF-β1. **b** as **a**, fractionation by ion exchange FPLC on a Mono Q^TM^ 5/50 G column. **c** Abundance of a candidate protein, Hp_I03161_IG00349_L1408, calculated by the exponentially modified Protein Abundance Index (emPAI) in each fraction, compared to the activation of TGFβ-responsive cells by the same fraction. **d** TGF-β bioassay screen of four candidate recombinant clones designated A–D; clone B corresponds to candidate Hp_I03161_IG00349_L1408 shown in panel **c**. Supernatants of cells transfected with clones A–D were assayed in duplicate, and mean values ± SEM are shown. Two-tailed *t* tests found Clone B to be significantly (*p* < 0.05) higher than all others. **e** Alignment of five similar domains within *Hp-*TGM encompassing the entire amino acid sequence apart from the predicted signal peptide (aa 1–18), with conserved cysteine (white on red) and other residues indicated, together with a Complement Control Protein (CCP) module from the nematode *Ascaris suum* (domain 12 of ASU_08405, aa 954–1018), and an archetypal CCP domain, human Factor H module 1 (X07523, aa 20–83). Other conserved residues are shown in red and potential *N*-glycosylation sites outlined in green. Amino acid positions for each domain of *Hp-TGM* are indicated on the left. Note the presence of a 15-aa insertion near the N-terminal of each domain of *Hp*-TGM which is not typical of the CCP family. Positions of disulfide bonds in Factor H are shown below the alignment by linked cysteine residues CI – CIV. **f** Exon-intron structure of *Hp*-TGM in the *H. polygyrus* genome; domains are colored corresponding to symbols in panel **e**; positions of cysteine residues indicated in black circles

To identify TGF-β-like activity, we screened *H. polygyrus* excretory-secretory products (HES) for their ability to activate the MFB-F11 fibroblast cell line in which an alkaline phosphatase reporter is activated by the Smad pathway upon receptor ligation^23^. HES proteins were independently fractionated by gel filtration and anion exchange Fast Protein Liquid Chromatography (FPLC), and each fraction assayed for activity on the reporter cell line (Fig. 1a, b). All fractions were then subject to mass spectrometric analysis for matching to a transcriptomic sequence database as previously described^21^. Eighteen proteins were identified for which abundance (measured by exponential mass protein abundance index, emPAI) was highest in the active fractions from both gel filtration and anion exchange (Supplementary Table 1); we selected 4 candidates to clone and express for which the abundance profile most closely matched biological activity in each fraction, as in the example shown in Fig. 1c, and in Supplementary Fig. 1.

For each candidate, mammalian codon-optimised sequences were synthesized and cloned into the plasmid vector pSecTag2a for transfection of human embryonic kidney HEK293 cells and expression as secreted recombinant proteins with hexa-histidine C-terminal tags. The supernatants of transfected cell cultures were collected and applied directly to the MFB-F11 assay. One transfectant (Hp_I03161_IG00349_L1408, the candidate shown in Fig. 1c), showed a high level of stimulatory activity, far exceeding that of total HES; this clone is depicted as clone B in Fig. 1d. From this clone, recombinant 49-kDa protein was expressed and purified by nickel chelating chromatography through affinity for the hexa-histidine tag. Following confirmation that the purified recombinant protein displayed TGF-β-like activity (see below), it was named *H. polygyrus* TGF-β Mimic *(Hp-*TGM).

The amino acid sequence of active *Hp-*TGM comprises 422 residues, of which the first 18 are predicted to form a classical signal peptide (Supplementary Fig. 2), with the remainder forming a mature 404-aa protein containing 22 cysteine residues (yellow on black) and 5 potential *N*-glycosylation sites (green). The protein has no sequence similarity to the TGF-β family in which the mature active moiety is a disulfide-linked homodimer of two ~ 110-aa C-terminal polypeptides with 6–9 cysteine residues. However, the mature protein of *H*p-TGM contains 5 homologous but non-identical ~ 80-aa domains each with distant similarity to the Complement Control Protein (CCP, or Sushi) family as marked by the positions of 4 cysteine residues and conserved tyrosine, glycine and tryptophan residues (Fig. 1e). Moreover, the mature protein is encoded in an 11-exon gene in the parasite genome, corresponding to the signal peptide (Exon 1) and 5 pairs of exons whose boundaries exactly match those of the CCP domains (Fig. 1f).

### *Hp*-TGM ligates TGF-β receptors

**Fig. 2:**
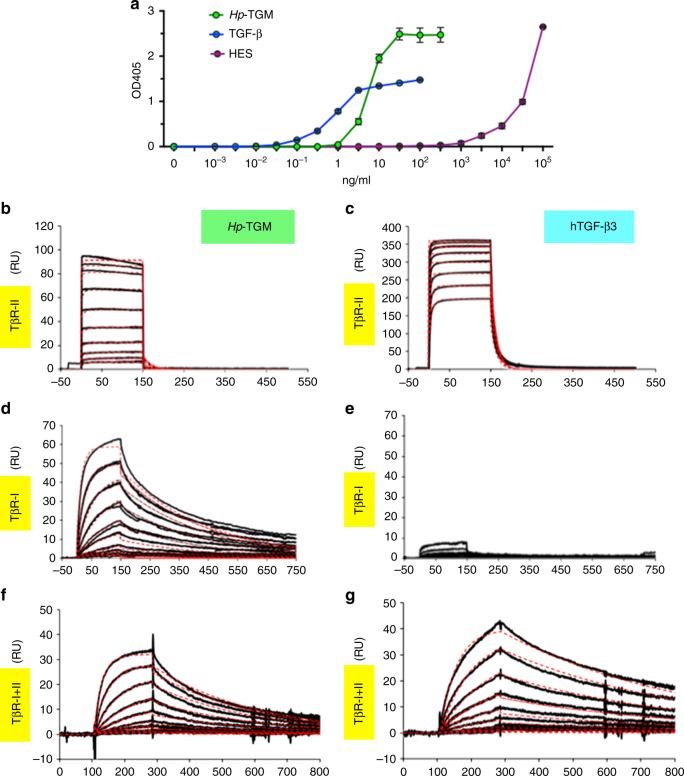
Binding of *Hp-*TGM to the TGF-β Receptors. **a** MFB-F11 TGF-β-responsive bioassay for activity following 24 h of culture at 37 °C, comparing *Hp-*TGM to hTGF-β1 and the complex HES mixture by protein concentration. MFB-F11 cells are transfected with a Smad-responsive plasmid construct in which TGF-β binding leads, through Smad phosphorylation and nuclear translocation, to expression of alkaline phosphatase, which is measured following the addition of *p*-nitrophenyl phosphate. Data shown are representative of  > 3 independent experiments, and represent mean ± SEM from duplicate wells. **b**–**e** Surface plasmon resonance analysis of *Hp-*TGM and hTGF-β3 binding to hTGF-β receptors. Streptavidin-coated biosensor chips were loaded with biotinylated *Hp*-TGM or hTGF-β3 and two-fold dilutions of the ectodomain of hTβRII (from 13 µM downwards) **b**, **c** and of the ectodomain of hTβRI (from 4 µM downwards) **d**, **e** were passed over the *Hp*-TGM or hTGF-β3 surface, respectively. Data shown are from one of two similar experiments. **f**, **g** Surface plasmon resonance analysis of *Hp-*TGM and hTGF-β3 binding to TβRI in the presence of near-saturating TβRII (2 μM), showing independent binding by *Hp-*TGM to both receptors, but binding of hTGF-β3 to TβRI dependent on the presence of TβRII

We next tested the ability of purified recombinant *Hp-*TGM protein (Supplementary Fig. 3) to activate MFB-F11 cells in vitro, in comparison to human TGF-β1 (hTGF-β1) and HES; all three induced reporter cell production of alkaline phosphatase in a dose-dependent manner (Fig. 2a). Notably, the primary *Hp-*TGM product proved to be active without the need for proteolytic processing to a mature form (as is required for mammalian TGF-β). The response of MFB-F11 cells to increasing concentrations of *Hp-*TGM reached a maximum signal significantly greater than attained by even the highest concentrations of hTGF-β1 (OD_405_ at 100 ng/ml, *Hp-*TGM = 2.46 ± 0.16 and hTGF-β1 = 1.48 ± 0.02, *p* = 0.02, multiple *t* test).

The MFB-F11 response to HES also exceeded the highest level of the TGF-β-induced signal, but required more than three log-fold higher concentrations to achieve the same signal as *Hp-*TGM (Fig. 2a). This indicated that *Hp-*TGM represents < 0.1% of the total protein present in HES, consistent with its low abundance ranking, from the mass spectrometric analysis even in the fractions that show peak TGF-β activity (Supplementary Table 1).

To ascertain whether *Hp-*TGM directly binds to TGF-β receptors (TβR), and to evaluate its binding affinity, recombinant protein was evaluated by surface plasmon resonance (SPR) with a sensor chip loaded with either *Hp*-TGM or human hTGF-β3; as shown in Fig. 2b–e, upon injection of hTβRII), this receptor bound *Hp*-TGM with lower affinity than hTGF-β3 (Fig. 2b, c), with a dissociation constant of 2.96 µM compared to 0.294 µM for hTGF-β3 (Supplementary Table 2). Remarkably, *Hp*-TGM bound directly to hTβRI with high affinity (Fig. 2d and Supplementary Table 2), in distinction to hTGF-β3, which alone binds hTβRI weakly (Fig. 2e)^24–26^. In contrast, binding of hTβRII had little to no effect on the binding of hTβRI to *Hp*-TGM (Fig. 2f), but it did strongly potentiate the binding of hTβRI to hTGF-β3 (Fig. 2g), as previously reported^24–26^ *. Hp-*TGM shows no binding affinity for TβRIII (also known as betaglycan), which is recognised by mammalian TGF-βs (Supplementary Fig. 4A).

**Fig. 3:**
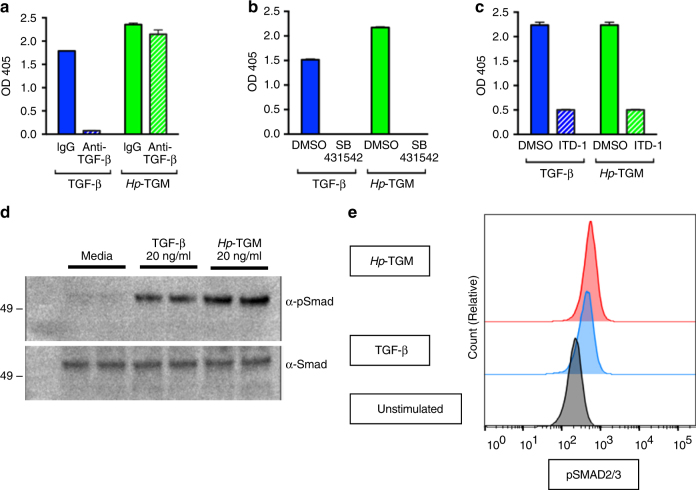
*Hp-*TGM signals through the TGF-β pathway. **a** Activity shown from MFB-F11 bioassay after 24 h of culture at 37 °C with hTGF-β1 or *Hp*-TGM incubated with anti-TGF-β monoclonal antibody or MOPC31C IgG control. Data shown are representative of two independent experiments, and represent mean ± SEM from duplicate wells; analysis by multiple *t* tests shows antibody significantly reduces effect of TGF-β (*P* < 0.0001), but has no significant effect on TGM. **b**, **c** Abolition of signaling by inhibitors of the TGF-β receptor kinases. Activity shown from MFB-F11 bioassay after 24 h of culture of TGF-β and *Hp-*TGM at 37 °C with: **b** the TβRI inhibitor, SB431542 or DMSO control and **c** the TβRII inhibitor, ITD-1 (10 µM). Data shown are representative of ≥ 2 independent experiments, and represent mean ± SEM from duplicate wells. Analysis by multiple *t* tests shows that effects of both mediators are significantly reduced by SB431542 (*p* < 0.0001) and ITD-1 (*P* < 0.001). **d** Western blots (Smad2 and phospho-Smad2): cell lysates from C57BL/6 splenocytes following culture at 37 °C for 18 h. Culture conditions in duplicate: media (DMEM + 2.5% FCS), media supplementted with 20 ng/ml hTGF-β1 and media supplemented with 20 ng/ml *Hp-*TGM. Position of a 49-kDa marker protein detected by negative staining is marked on each blot. **e** Phospho-Flow analysis with anti-phospho-Smad2/3 on murine CD4^+^ T cells stimulated for 16 h with 20 ng/ml of *Hp-*TGM or hTGF-β1, before permeabilization and staining with specific antibody. A representative individual histogram is displayed

We then established if *Hp-*TGM signaling could be inhibited by antibody to mammalian TGF-β, to exclude the possibility that the parasite protein interacts with or activates the host cytokine in tissue culture medium. MFB-F11 cells were first co-cultured with *Hp-*TGM or hTGF-β1 and 100 μg/ml pan-vertebrate anti-TGF-β antibody (clone 1D11) or MOPC murine IgG control. Anti-TGF-β antibody considerably inhibited the MFB-F11 signal generated from hTGF-β1, but had no impact on *Hp-*TGM (Fig. 3a). Consistent with these findings, 1D11 antibody bound only hTGF-β3 and not *Hp*-TGM by SPR (Supplementary Fig. 4B), while polyclonal rat anti-*Hp*-TGM IgG bound only *Hp*-TGM and not hTGF-β3 (Supplementary Fig. 4C).

To establish if *Hp-*TGM transduces canonical signalling following TGF-β receptor ligation, the MFB-F11 cells were stimulated in the presence or absence of inhibitors of receptor kinase activity. We first tested the kinase inhibitor SB431542 which blocks phosphorylation by TβRI, as well as other TGF-β family type I receptors Alk5 and Alk7^27^. SB431542 has previously been found to block the TGF-β-like activity of unfractionated HES, and to render mice more resistant to *H. polygyrus* infection^9^. Both *Hp-*TGM and hTGF-β1 signals were completely ablated in the presence of SB431542 (Fig. 3b). We repeated this assay with “Inducer of Type II TGF-β Receptor Degradation-1” (ITD-1)^28^, which also completely ablated the MFB-F11 signal generated by *Hp-*TGM and hTGF-β1 (Fig. 3c), indicating that both ligands directly signal through the same combination of type I and II receptors on mammalian cells.

Further evidence that *Hp-*TGM can signal through the TGF-β pathway was found when stimulating splenocytes from C57BL/6 mice. Following overnight incubation with saturating concentrations of each ligand, cells were collected and assayed for Smad2/3 phosphorylation by Western blotting and flow cytometry. As shown in Fig. 3d, e, *Hp-*TGM induces phosphorylation of Smad2/3 phosphorylation at least as effectively as TGF-β). In contrast, no activation of either the Akt pathway (Supplementary Fig. 5A) or the p38 pathway (Supplementary Fig. 5B) was evident with either host or parasite ligand.

### Treg cell induction by *Hp*-TGM

**Fig. 4:**
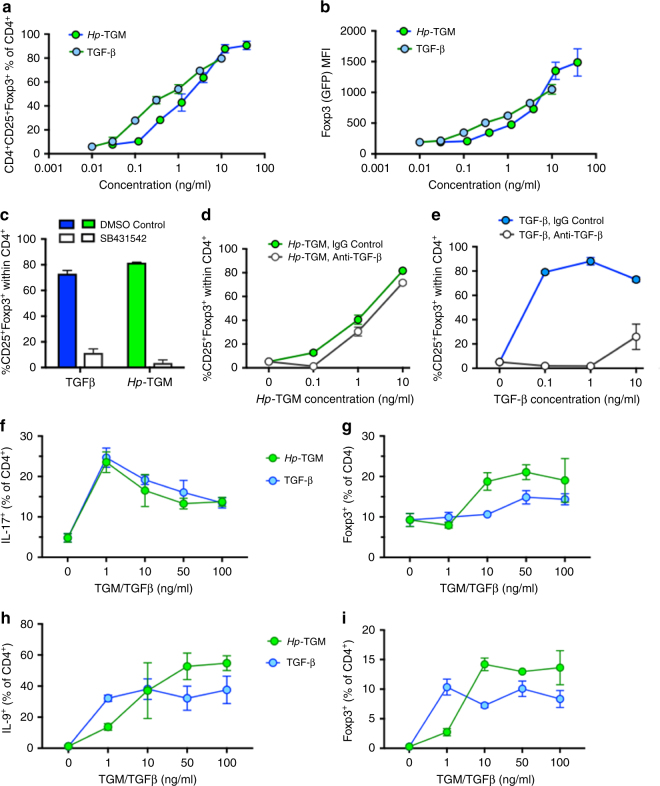
*Hp-*TGM induces T cell Foxp3 expression even in proinflammatory conditions. **a**, **b** CD4^+^CD25^–^GFP^–^CD62L^hi^ murine naive T cells were stimulated with plate-bound anti-CD3/CD28 for 4 days in culture with 100 U/ml IL-2 and variable concentrations of *Hp-*TGM or hTGF-β1, before flow cytometric analysis of CD4, CD25 and Foxp3 expression; 2 technical replicates per concentration; representative of 4 independent experiments;. **a**: percentage of CD25^+^Foxp3^+^ cells among total CD4^+^ cells; **b**, Mean fluorescence intensity (MFI) of Foxp3 among Foxp3^+^ cells. Gating strategy is shown in Supplementary Fig. 6. **c**–**e** Foxp3 induction in the same conditions as **a**, in the presence of SB431542 inhibitor **c** or pan-vertebrate anti-TGF-β **d**, **e**; 2 technical replicates per concentration; representative of 3 independent experiments. Analysis by multiple *t* tests showed no significant difference in responses to TGM when anti-TGF-β was present, but signicant effects (*p* < 0.01) on responses to all concentrations of TGF-β. **f**, **g** Magnetically sorted murine CD4^**+**^ T cells were cultured for 5 days with Th17-promoting ligands (100 ng/ml IL-6, 5 ng/ml IL-23, 10 ug anti-IFN-γ), together with the indicated concentrations of *Hp*-TGM or TGF-β, then stimulated with PMA/ionomycin in the presence of Brefeldin A for 4.5 h before staining for intracellular Foxp3 and IL-17. Data are mean ± SEM of triplicate replicates from 1 of 2 independent experiments; data were analysed by multiple *t* tests which showed no significant difference at any concentration of TGF-β v TGM for percentage IL-17^+^, and *p* < 0.001 and *p* < 0.01, respectively for 10 and 50 ng/ml TGM v TGF-β for percentage Foxp3^+^. Gating strategy is shown in Supplementary Fig. 6. **h**, **i** Murine CD4^**+**^ T cells were cultured as above with but with Th9-promoting ligands (40 ng/ml IL-4, 20 ng/ml IL-2), and assayed as above after 4 days of culture by staining for intracellular Foxp3 and IL-9. Data are mean ± SEM of three replicates from 1 of 2 independent experiments; data were analysed by multiple *t* tests which showed *p* < 0.05 for 1 ng/ml TGF-β v TGM for percentage IL-9^+^, and *p* < 0.001 for 10, 50, and 100 ng/ml TGM v TGF-β for percentage Foxp3^+^. Gating strategy is shown in Supplementary Fig. 6

*H. polygyrus* and HES have previously been shown to induce Foxp3^+^ Treg cells in vitro and in vivo^8, 9, 29^. We therefore next ascertained if *Hp-*TGM could induce Foxp3^+^ Treg cell differentiation in vitro and if the evidence of potent intracellular signalling would be reflected in the level of Foxp3 expression within the induced Treg population. CD4^+^CD25^–^GFP^–^CD62L^hi^ cells were isolated from Foxp3-GFP reporter mice^30^ by MACS and FACS sorting, then cultured for 96 h in Treg polarising conditions with hTGF-β1 or *Hp-*TGM. *Hp-*TGM was found to effectively induce Foxp3^+^ Treg cell differentiation: at the highest concentration tested (38.1 ng/ml, 0.78 nM) *Hp-*TGM induced Treg cell conversion in 90.65% (±3.55%) of all CD4^+^ cells, compared to 79.65% (±2.55%) induced by a similar molar concentration (10 ng/ml, or 0.39 nM homodimeric hTGF-β1, effectively 0.78 nM monomer concentration) of the human cytokine (Fig. 4a). Further, the mean fluorescence intensity of Foxp3 expression induced by high concentrations of *Hp-*TGM was found to be greater than that of the equivalent concentration of hTGF-β1 (Fig. 4b). *Hp-*TGM-mediated induction of Foxp3 was completely abolished by the TGF-βRI kinase inhibitor SB431542 (Fig. 4c), but not by pan-vertebrate anti-TGF-β antibody (Fig. 4d), while the presence of either reagent blocked the effect of the mammalian ligand (Fig. 4c, e).

In the presence of other cytokines such as IL-6 and IL-4, TGF-β is known to promote in vitro differentiation of effector T cell subsets designated as Th17^31^ and Th9^32^ respectively. We therefore evaluated whether *Hp*-TGM exhibits corresponding activity on purified murine CD4 + T cells. In the presence of IL-6, both TGF-β and *Hp*-TGM induced similar levels of Th17 development as measured by intracellular staining of IL-17, particularly at lower ligand concentrations (Fig. 4f). However, under the same conditions, *Hp-*TGM was able to drive a significant expansion of Foxp3^+^ Treg cells, which at higher ligand concentrations even outnumbered the Th17 population (Fig. 4g) and few of which co-expressed IL-17 (data not shown). In the case of Th9-favouring conditions including IL-4, TGF-β and *Hp*-TGM elicited broadly similar outcomes in terms of IL-9^+^ T cells (Fig. 4h), although again a significant induction of Foxp3^+^ Treg cells was also observed particularly in the presence of *Hp*-TGM (Fig. 4i).

**Fig. 5:**
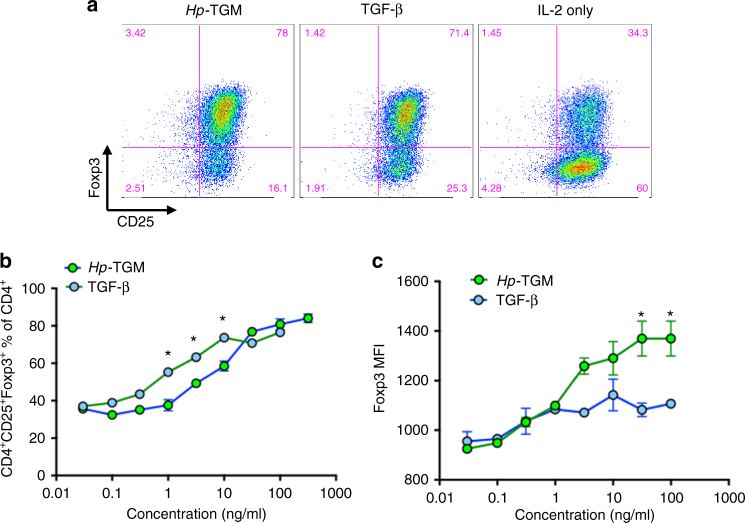
*Hp*-TGM induces Foxp3 expression in human T cells. **a**–**c** Human peripheral blood mononuclear cells were separated from red blood cells over a Ficoll gradient and CD4^+^ T cells isolated by MACS positive selection. Isolated cells were cultured at 37 °C for 96 h with a 1:1 ratio of CD3/CD28 Dynabeads® and variable concentrations of hTGF-β1 or *Hp-*TGM. Induction of Tregs from human peripheral blood monuclear cells. Data are means and SEM from two technical replicates per concentration and representative of 2 independent experiments, analysed by unpaired *t* test and corrected for multiple comparisons; *p < 0.05. Gating strategy is shown in Supplementary Fig. 6. **a** Representative flow cytometry plots (CD4^+^ population shown) of MACS-purified CD4^+^ positive selected PBMCs stimulated with *Hp*-TGM, hTGF-β or IL-2 respectively; **b** Percentage of CD25^+^Foxp3^+^ cells among total CD4^+^ cells; **c** MFI of Foxp3 among Foxp3^+^ cells

As *Hp-*TGM was found by SPR to bind to the human TβRs, we then tested whether it could drive expression of Foxp3 in human CD4^+^ T cells purified from peripheral blood cultured with anti-CD3/CD28 Dynabeads and variable concentrations of *Hp-*TGM or hTGF-β1 for 96 h before assessment of CD25 and Foxp3 expression. As shown in Fig. 5a, b the proportion of CD4^+^CD25^+^Foxp3^+^ Treg cells increased with *Hp*-TGM in a concentration-dependent fashion, to a maximum of 84% (±2.5%). The proportion of Treg cells within all CD4^+^ cells was similar for *Hp-*TGM and hTGF-β1 at most concentrations; however, at higher concentrations, the mean fluorescence intensity (MFI) of Foxp3 expression was significantly greater in Treg cells exposed to *Hp-*TGM compared to hTGF-β1 (Fig. 5c).

**Fig. 6:**
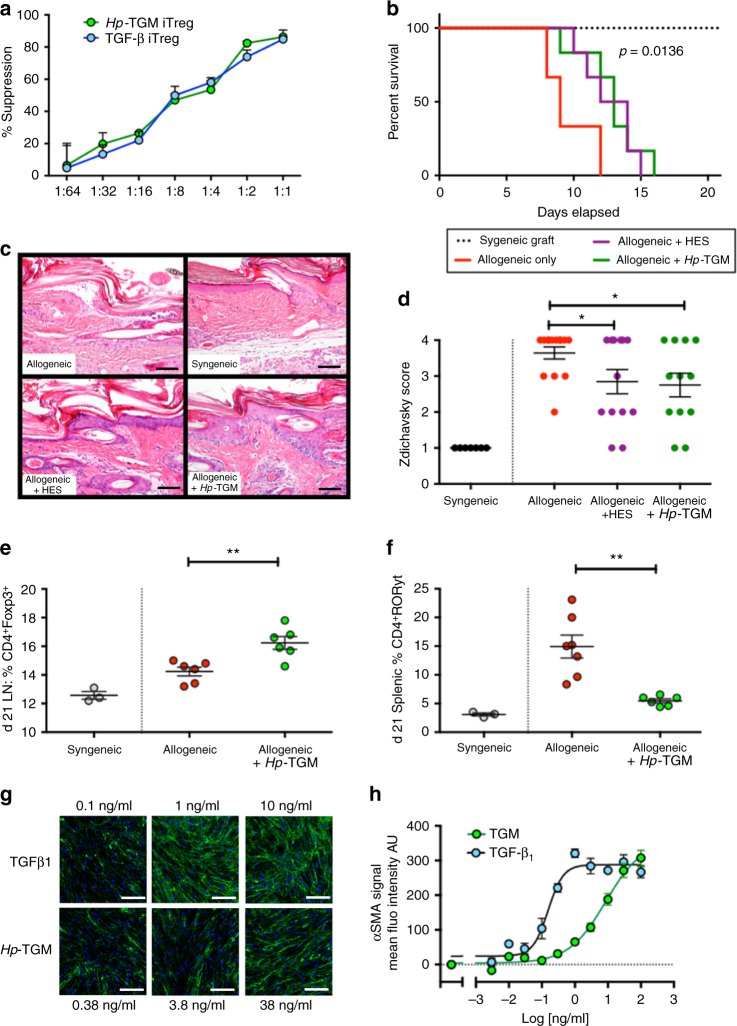
*Hp*-TGM drives immune regulation in vitro and in vivo. **a** Mouse Foxp3^+^ Treg cells are suppressive in vitro. CD4^+^CD25^+^GFP^+^ cells, generated as Fig. 4a were isolated by FACS and co-cultured with CD4^+^CD25^–^GFP^–^ responder cells from Foxp3-GFP mice together with, irradiated APCs and anti-CD3 for 5 days. Proliferation was assessed by thymidine incorporation, and percentage suppression calculated; data are means ± SEM from 3 replicates per concentration and represent two independent experiments. **b** *Hp-*TGM prolongs survival of fully allogeneic full-thickness BALB/c to C57BL/6 skin grafts. Kaplan–Meier curve of graft survival: allograft only (*n* = 6), allograft + HES or *Hp-*TGM following implantation of intraperitoneal minipumps, *n* = 6) and syngeneic controls, *n* = 3. Mantel-Cox comparison of allograft vs. allograft + *Hp-*TGM survival curves: *p* = 0.0136. **c**,** d** Histological analyses of graft sites 7 days following transplantation; **c**, representative images of tissues sections, scale bars = 100 µm; **d**, scoring of inflammation on 3 sections per graft performed in a blinded fashion: syngeneic control (*n* = 7), allograft + control protein (*n* = 14), allograft + HES (*n* = 13), allograft + TGM (*n* = 12); data shown combine two independent experiments, with mean ± SEM shown. HES vs untreated allogeneic control *p* = 0.0493; *Hp*-TGM v untreated control *p* = 0.0397, by unpaired *t* test. **e**,** f** Treg and Th17 CD4^+^ T cell populations 21 days after transplantation, in **e** draining lymph node Foxp3^+^ (syngeneic controls (*n* = 3), allograft + control protein (*n* = 6), allograft + TGM minipump (*n* = 6); two-tailed, unpaired *t* test: *p* = 0.0042) and **f** spleen RORγt^+^ (syngeneic controls (*n* = 3), allograft + control protein (*n* = 7), allograft + TGM minipump (*n* = 6); two-tailed, unpaired *t* test: *p* = 0.0112). Gating strategy is shown in Supplementary Fig. 6. **g**,** h** Fibrogenesis in human lung fibroblasts exposed to TGFβ1 or *Hp-*TGM, assayed by immunofluoresence for collagen deposition (**g**, see Supplementary Fig. 8A for quantitative summary) and smooth muscle actin (**h**, presented as quantitative summary, see Supplementary Fig. 8B for fluorescence micrographs). Human collagen I stained with AF488-conjugated scondary antibody (green) and counterstained with DAPI (blue). Scale bars = 200 µm. Data are means ± SEM from 1 of 3 replicate experiments, with 4 technical replicates per group

To further establish if *Hp-*TGM can induce immune suppressive function in naïve T cells, murine Treg cells generated from sorted CD4^+^CD25^–^GFP^–^CD62L^hi^ cells incubated with hTGF-β1 and *Hp-*TGM were added to CD4^+^CD25^−^GFP^−^CD62L^hi^ responder cells together with soluble anti-CD3 and irradiated APC. Assessment of responder cell proliferation by thymidine incorporation demonstrated that *Hp-*TGM-generated Treg are functionally suppressive in vitro with suppressive capacity equivalent to TGF-β-generated Treg cells (Fig. 6a).

### Anti-inflammatory effects of *Hp*-TGM

To test the efficacy of *Hp*-TGM in an in vivo model of immunopathology, we examined its effects in a model of allograft rejection, as helminth parasites and their products have been previously described to prolong the life of tissue transplants^33^. We chose the fully allogeneic skin transplant model, from BALB/c donor mice to C57BL/6 recipients, a system in which median rejection occurs in ~9 days, mediated by Th1 and Th17 inflammatory responses. This represents a robust and intense allogeneic reaction^34–36^ but one which is known to be down-modulated by adoptive transfer of Treg populations^37^. *Hp-*TGM or HES were administered to mice through osmotic mini-pumps inserted intraperitoneally to continuously release parasite products in a manner akin to live infection. Both *Hp-*TGM and HES conferred a significant protraction of allograft life, with median survival extended by ~ 5 days relative to untreated controls (Fig. 6b); it is worth noting that such extension reflects a very effective immune modulation comparable with, for example, the recent use of mesenchymal stem cells in this model^38^.

In parallel experiments, inflammation at the graft site was assessed 7 days post-transplant in animals treated with HES or *Hp*-TGM, in comparison to controls. As shown in Fig. 6c, both treatments reduced histological features of rejection such as dermal inflammation and epidermal degeneration. Sections were evaluated blindly using the Zdichavsky scoring system^39^, revealing significantly attenuated rejection reactions in recipients of both HES and *Hp-*TGM (Fig. 6d).

In allograft recipients exposed to *Hp-*TGM, a significant increase in Foxp3^+^ expression was observed in the allograft draining lymph node (Fig. 6e) and spleen (Supplementary Fig. 7A) at day 21. Moreover, the expression of T cell RORγt, indicative of Th17 expansion induced by the allograft, was reduced in recipients of *Hp-*TGM to the level observed in syngeneic graft recipients (Fig. 6f), as was expansion of Tbet^+^ Th1 cells (Supplementary Fig. 7B). In separate experiments, when lymphoid tissues were sampled 7 days following allografting, similar reductions of inflammatory cell phenotypes were observed, including diminished T-bet expression among total CD4^+^ T cells (Supplementary Fig. 7C).

Mammalian TGF-β is a multi-faceted molecule with effects beyond the regulatory T cell circuit, which can contraindicate its therapeutic use as an immunosuppressant or anti-inflammatory agent. Within the T cell compartment, TGF-β is associated with Th17 differentiation when IL-6 is present in vitro^31, 40^, or when TGF-β is over-expressed in Freund’s complete adjuvant-immunized mice^40^; the suppression of RORγt expression by *Hp-*TGM in allogeneic skin graft recipients suggests that the parasite ligand does not potentiate Th17 responses in vivo, while our in vitro assays indicate that *Hp-*TGM can still induce significant regulatory T cell development even in a nominally pro-inflammatory environment (Fig. 4f–i). A second potential therapeutic limitation of TGF-β arises from its ability to induce fibrosis^41^, promote myofibroblast differentiation and excessive deposition of extracellular matrix. Importantly, we found that *Hp*-TGM is significantly less fibrogenic than mammalian TGF-β in terms of promoting fibroblast collagen I deposition (Fig. 6g, Supplementary Fig. 8A) and alpha-smooth muscle actin induction (Fig. 6h, Supplementary Fig. 8B) in human lung fibroblasts. Finally, a caveat of applying xenogeneic proteins in a therapeutic context in vivo is the potential generation of neutralizing antibodies in the recipient; this may militate against protocols requiring repeated administration of *Hp-*TGM in vivo, but would not exclude ex vivo applications, for example conversion of patients’ T cells in vitro into the regulatory phenotype prior to return to the autologous individual.

## Discussion

Helminth parasites are now well-known to exploit the immunoregulatory power of the TGF-β pathway, driving the production of this cytokine by host cells, and promoting longer-term establishment of the parasite in mammalian tissues. Many pathogens, particularly viruses, imitate host cytokines, or even express cytokine genes originally captured from the genome of their host^42^. However, no previous example has been reported of a completely unrelated structural product elaborated by parasites that so closely mimics the activity of a crucial host cytokine. Furthermore, the imitation of TGF-β is itself striking, as this mediator is the single most immune-suppressive and pro-tolerogenic product of the host immune system involved in a suite of critical immunoregulatory pathways^43^. The ability of TGF-β to induce and expand suppressive Treg cells is arguably the most prominent immunological function of TGF-β^12, 44, 45^, while Treg cells have been shown to be essential for the survival of several helminth parasites in vivo^4, 5^ including *H. polygyrus* ^10^.

As metazoa, helminth organisms also encode endogenous members of the TGF-β ligand and receptor families, which in some settings can interact with cognate partners of vertebrate origin^46–50^. Further, we had reported that immunomodulatory Treg cells were induced by *H. polygyrus* secreted products acting through the TGF-β pathway^9^. We now identify the molecular agent responsible, and find that rather than belonging to the classical TGF-β family, the parasite molecule represents an unexpected and novel structure. This finding emphasizes the remarkable immunomodulatory strategy of *H. polygyrus* which has convergently evolved, from the scaffold of the CCP family, a unique multi-domain structure able to signal through the TGF-β pathway and, like TGF-β itself, induce potently suppressive Treg cells and abate inflammation in vivo.

Despite ligating the TGF-β signaling receptors, TβRI and TβRII, and driving Smad phosphorylation, *Hp-*TGM is structurally distinct from the TGF-β molecule and evidently binds TβRI and TβRII sites that are well-separated, and not directly adjacent to one another as in the TGF-β receptor complex^24, 25^. *Hp-*TGM shares no sequence homology with TGF-β, is almost twice the size of a TGF-β homodimer (49 kDa vs. 25 kDa), and is not recognised by pan-vertebrate anti-TGF-β antibodies. Furthermore, *Hp-*TGM is constitutively active, in contrast to the TGF-β ligands which are processed from a longer pro-protein to a mature ~ 110-aa growth factor domain by proteolytic cleavage at a conserved furin site (RRXR)^51^.

*Hp*-TGM is a member of the CCP superfamily defined by a 60–80 aa domain with 4 cysteines and key characteristic residues including a conserved tryptophan. In most eukaryotic species, including nematodes, the protein family has expanded and radiated with extensive diversity of structure and function. Interestingly, searching the genomes of the human hookworms *Necator americanus* ^52^ and *Ancylostoma duodenale* reveals 12–18 members of this gene superfamily, each with low levels of sequence similarity to *Hp*-TGM. It remains to be determined if one or more of these homologues may share the cytokine-like activity of *Hp-*TGM.

A notable finding has been that *Hp-*TGM stimulates greater expression of Foxp3 compared to that achieved with TGF-β in both murine and human CD4^+^ T cells. Intensity of Foxp3 expression by Treg cells has previously been shown to directly correlate with suppressive ability^53^ while high concentrations of TGF-β favour Treg over Th17 differentiation^54^. Further studies are required to ascertain if *Hp-*TGM is able to deliver a stronger signal through the canonical TGF-β receptor cascade, or is perhaps inured to inhibitory pathways that naturally counteract signals from the mammalian ligand such as the pseudoreceptor BAMBI^55^.

Within the inducible Treg compartment, expression of Foxp3 and consequent regulatory function is in some settings reversible^56, 57^. We are now studying whether *Hp*-TGM drives a more stable and longer-lasting Treg phenotype, and/or one less influenced by inflammatory cytokines such as IL-6. If so, then new therapies such as autologous transfusion following ex vivo expansion of Treg cells^58^ may be much enhanced in terms of both efficacy and safety.

The ability of *Hp-*TGM to delay allograft rejection, and to inhibit all three major subsets of effector CD4^+^ T cells in vivo, also portends well for a therapeutic application of this new molecule. Recombinant *Hp-*TGM offers several advantages including scalable production, a definable mechanism of action and the opportunity for modification to reduce immunogenicity and optimise pharmacokinetic characteristics for pharmacological use. Furthermore, combinations of *Hp-*TGM with currently available immunomodulatory agents may further enrich future therapeutic strategies in which the directed manipulation of the different T cell subsets will offer resolution of inflammatory conditions of diverse aetiologies.

## Methods

### Mice

Inbred female C57BL/6 J OlaHsd, BALB/c OlaHsd, and Foxp3-GFP reporter mice^30^ were used for experiments, aged 6–12 weeks old, bred in-house or purchased from Harlan Laboratories, and maintained in individually-ventilated cages. All animal experiments were performed under UK Home Office licence and approved by the University of Edinburgh and/or University of Glasgow Ethical Review Board(s). No randomization was used and no animals were excluded from analysis.

### Fractionation of HES and mass spectrometric analysis

*Heligmosomoides polygyrus* Excretory Secretory (HES) products were prepared as described elsewhere^59^. HES was separated into 1 ml fractions using an ÄKTApurifier^TM^ (GE Healthcare) using either the Superdex 200 10/300 GL column (GE Healthcare) for gel filtration fractionation or the Mono Q^TM^ 5/50 GL (GE Healthcare) for anion exchange fractionation. The protein concentration of fractions was measured by Pierce BCA Protein assay kit (Thermo Scientific) with 5 μg of each fraction being trypsin digested, analysed using an Orbitrap mass spectrometer and then compared to an in house *H. polygyrus* transcriptomics database using the Mascot program set with default parameters (Matrix Science), to generate a Protein Score and a probability (*p*) that a match would occur by chance; proteins calculated by the Mascot program to return a score below 20 or *p* > 0.05 were not considered. Scripts written in Python 2.7 were used to analyse the mass spectrometry results.

### Recombinant *Hp*-TGM

Recombinant *Hp-*TGM was synthesised as a mammalian codon optimised insert (GeneArt) and cloned into the mammalian expression vector pSECTag2a (Invitrogen). The construct was transfected into HEK293T cells using the calcium chloride transfection method (Promega) and recombinant *Hp-*TGM was purified from culture supernatant by Ni-chelating chromatography (Supplementary Fig. 2).

### Antibodies and inhibitors

The mouse monoclonal pan-isoform specific TGF-β IgG antibody, 1D11^60^, was purchased (BioXCell), as were the inhibitors SB431542 (Tocris Bioscience) and ITD-1 (Tocris Bioscience). The mouse IgG1 myeloma MOPC 31 C from ATCC (ECACC-90110707) was used as an isotype control. Smad2/3 antibody (Cell Signaling Technology) and phosphoSmad2 antibody (Cell Signaling Technology) was used as the primary antibody for Western blots, followed by Goat anti rabbit IgG-HRP secondary (BioRad) according to manufacturer’s protocols. Recombinant *Hp*-TGM was detected by Western blot using an anti penta-His-HRP conjugate (Qiagen). An uncropped image of the Western blot shown in Fig. 3d is presented in Supplementary Fig. 9.

### TGF-β bioassay

The TGF-β bioassay (MFB-F11) developed by Tesseur et al.^23^ was used with embryonic fibroblasts from TGF-β1^–/–^ mice stably transfected with a TGF-β-responsive reporter plasmid containing a secreted embryonic alkaline phosphatase reporter gene (SBE-SEAP). MFB-F11 cells were grown in DMEM with 10% FCS, 100 U/ml penicillin, 100 μg/ml streptomycin, 2 mM L-glutamine and supplemented with 15 μg/ml Hygromycin B (Invitrogen), for 3 days. Cells were tested and found to be mycoplasma-free. Confluent cells were detached with trypsin, and resuspended in DMEM with 2.5% FCS, 100 U/ml penicillin, 100 μg/ml streptomycin and 2 mM L-glutamine at a concentration of 4 × 10^5^ cells/ml. In 50 μl, 4 × 10^4^ cells were added to each well of a 96-well round-bottomed plate. Serial dilutions of test substances HES (in-house), *Hp-*TGM (in-house), and recombinant human TGF-β1 (R&D Systems) were then added to each well in a volume of 50 μl and incubated for 24 h at 37 °C. Subsequently, 20 μl of supernatant was aspirated from each well, added to an ELISA plate (NUNC) with 180 μl of reconstituted Sigma FastTM p-nitrophenyl phosphate substrate and incubated at RT in the dark for up to 4 h. Plates were read on at 405 nm on an Emax precision microplate reader (Molecular Devices).

### Recombinant proteins for surface plasmon resonance

The human TGF-β type I and type II receptor extracellular domains, hTβRI and hTβRII, were produced as insoluble proteins in *E. coli* and reconstituted, refolded, and purified to homogeneity as previously described^61, 62^. The rat TGF-β type III receptor extracellular domain, which is also known as rTβRIII or r-betaglyan, was produced in cultured HEK293 freestyle cells (Invitrogen) and purified to homogeneity using metal affinity chromatography and size exclusion chromatography as described elsewhere^63^. Human TGF-β3 (hTGF-β3), and human TGF-β3 bearing an N-terminal 15 amino acid Avitag^64^ followed by an EY linker (Avi-hTGF-β3), were produced in *E. coli*, reconstituted in urea, refolded, and purified to homogeneity using ion-exchange chromatography, as previously described^65^. The mouse monoclonal pan-isoform specific TGF-β IgG antibody, 1D11^60^, was purchased (BioXCell), whereas the rat polyclonal anti-*Hp*-TGM antibody was produced in-house by immunization with 10 µg protein in alum adjuvant on days 0, 28, and 35, with serum recovered on day 42.

Biotinylated Avi-hTGF-β3 for surface plasmon resonance (SPR) studies was generated by complexing Avi-hTGF-β3 with r-betaglycan in 10 mM bicine at pH 8.0 and then biotinylated by incubating with a catalytic amount of bacterially expressed BirA recombinase, biotin, magnesium, and ATP at 37 °C for 2 h, as described^64^. Biotinylated Avi-hTGF-β3 was then bound to a C4 reverse phase column equilibrated with 94.9% water/5% acetonitrile/0.1% triflouroacetic acid and eluted with a linear acetonitrile gradient. Attachment of a single biotin to each protein chain was confirmed by measuring the intact mass of the biotinylated, purified proteins using electrospray ionization-time of flight mass spectrometry (Agilent). *Hp*-TGM was biotinylated using EZ-Link Sulfo-NHS-Biotin (Thermo Scientific) according to the manufacturer’s protocol.

### SPR

SPR was used to assess the binding of hTβRI, hTβRII, and r-betaglycan) to *Hp*-TGM and hTGF-β3. SPR was also used to validate the *Hp*-TGM and hTGF-β3 surfaces using a rat polyclonal *Hp*-TGM antibody and mouse monoclonal pan-isoform specific TGF-β antibody, 1D11^60^. A BIAcore 3000 SPR instrument (GE Lifesciences) was used for studying antibody, betaglycan, and TβRII binding with streptavidin-coated carboxy methyl dextran (CM5) sensor chips (GE Lifesciences) with biotinylated Avi-hTGF-β3 or *Hp*-TGM captured at a surface density of 400–500 resonance units (RU). A BIAcore × 100 instrument (GE Lifesciences) was used for studying hTβRI binding, either in the absence or presence of 2 μM hTβRII, with hTGF-β3 and *Hp*-TGM covalently attached to a carboxymethylated dextran sensor chip (CM5, GE Lifesciences) by carbodiimide-based amine coupling. Coupling of hTGF-β3 and *Hp*-TGM to the sensor chip was performed using an amine coupling kit (GE Lifesciences) according to manufacturer’s protocol.

SPR binding assays were performed by injecting two-fold serial dilutions of analytes in HBS-EP buffer (GE Healthcare) at a flow rate of 100 µL min^−1^ (*Hp*-TGM antibody, 1D11 antibody, r-betaglycan, or hTβRII) or 30 µL min^−1^ (hTβRI) over the hTGF-β3 and *Hp*-TGM surfaces. hTβRI binding was also investigated in the same manner, but with 2 mM hTβRII included in both the HBS-EP running buffer as well as the injected hTβRI samples. All injections were performed at room temperature and were preceded by a brief injection of 4 M guanidine hydrochloride for 30 s to regenerate the surface. Baseline correction was performed by double referencing^63^. Kinetic analyses were performed by global fitting with a simple 1:1 model using the Biaevaluation software (GE Lifesciences).

### Cellular immunology assays

Single cell suspensions were made from murine spleen and lymph node specimens by maceration through 70 μm filters (BD) into complete RPMI 1640 (cRPMI) medium containing HEPES (Gibco), supplemented with 2 mM L-glutamine, 100 U/ml penicillin and 100 μg/ml streptomycin (Gibco), 10% heat-inactivated foetal calf serum (FCS) (Gibco), and 50 nM 2-mercaptoethanol (Gibco). Contaminating red blood cells were removed by resuspending the cells from one spleen in 2 ml of red blood cell lysis buffer (Sigma) and incubating at RT for 2 min. Cells were then washed with cRPMI and counted on a haemocytometer by trypan blue exclusion. For human lymphocytes, fresh peripheral blood was obtained by venepuncture of healthy volunteers under a protocol approved by the University of Edinburgh research ethics committee. Blood was collected into heparinised tubes (BD) and immediately diluted 1:1 with PBS, centrifuged over Ficoll-Paque (GE Healthcare) at 400×*g* for 40 min at RT with no brake, and PBMCs recovered from the interface before three further washes in cRPMI at 200 g for 10 min (RT). Finally, cells were counted on a haemocytometer in preparation for culture.

### Flow cytometric analysis and cell sorting

For viability staining, LIVE/DEAD® fixable blue (Life Technologies) was diluted to 1:1000 in PBS; 200 μl was added to each sample of cells, which were then incubated in the dark for 20 min at 4 °C (protected from light) and washed twice in FACS buffer. To prevent non-specific antigen binding, cells were incubated with 50 µl of polyclonal IgG (diluted 1:50 in FACS buffer) for 10 min at 4 °C and then washed twice in FACS buffer. All samples were acquired on a BD Biosciences LSR II or LSR Fortessa flow cytometer and analysed using FlowJo software (Tree Star).

The following FACS antibodies were diluted to an appropriate final concentration in FACS buffer (or permeabilisation buffer (eBioscience) for intracellular antibodies): Anti-CD3-FITC (17A2, Biolegend, 1/200); anti-CD4-AF700 and –BV650 (RM4-5, Biolegend, 1/200); anti-CD8-PerCP (53–6.7, Biolegend, 1/200); anti-CD25^−^APC (PC61-5, eBioscience, 1/200); anti-Foxp3-ef450, (FJK-16s, eBioscience, 1/50), anti-ROR-gamma(t)-PE (AFKJS-9, eBioscience, 1/50); anti-Tbet-PerCP-Cyanine (eBio4BIO, eBioscience, 1/50) to a total volume of 50 μl diluted antibody per 5 × 10^6^ cells. Single stain controls were individually added to one drop of UltraComp eBeads (eBioscience). Samples were incubated for 20 min at 4 °C, washed twice in FACS buffer and then resuspended in 200 μl FACS buffer for acquisition of surface marker data directly or further processed for intracellular staining.

### CD4^+^ T cell enrichment by magnetic sorting

Cells were resuspended in MACS buffer at a volume of 45 μl per 10^7^ cells, together with 5 μl of microbeads (L3T4, Miltenyi Biotech), and incubated at 4 °C for 20 min. Cells were then washed three times in MACS buffer, centrifuging at 200×*g* for 5 min, and resuspended in MACS buffer at a volume of 50 μl per 10^7^ cells. CD4^+^ cells were then isolated by performing a positive selection using an AutoMACS (Miltenyi Biotech) automated magnetic column as per the manufacturer’s instructions. The positive fraction of cells was then resuspended in MACS buffer and counted.

### Fluorescence-activated cell sorting

CD4^+^ cells (freshly isolated or from culture) were enriched by magnetic sorting as above and then incubated with antibodies for surface markers as described above, but with the omission of a viability stain. Following staining, cells were resuspended in MACS buffer at a concentration of 5 × 10^8^ cells per ml. Sorting was performed on a BD FACSAria with a gating strategy of: lymphocytes (by forward and side scatter), single cells and then stained populations, e.g., CD4^+^CD25^–^Foxp3^–^CD62L^hi^. Cells were sorted into 2 ml of FCS (Gibco) and a sample from each tube was re-acquired on the FACSAria to assess the purity of each sort.

### T cell polarization

To provide conditions for TH9 and Th17 cell polarization, CD4^+^ T cells enriched by magnetic sorting were cultured at 1 × 10^5^ per well in flat-bottomed 96-well plates (Costar) pre-coated with 2 μg/ml αCD3 (eBioscience) and 2 μg/ml or 1 μg/ml αCD28 (eBioscience), for Th9 and Th17 conditions respectively. Cytokines for Th9 conditions were added in complete medium as follows 40 ng/ml IL-4, 20 ng/ml IL-2 and for Th17 conditions 100 ng/ml IL-6, 5 ng/ml IL-23 (all from Miltenyi Biotech) and both polarization cultures contained 10 μg/ml αIFN-γ (BioXcell) in addition to varying concentrations of hp-TGM or hTGF-β3. Cultures were left at 37 °C with 5% CO_2_ and restimulated with 500 ng/ml PMA, 500 ng/ml ionomycin and 1 µg/ml BFA (Sigma Aldrich) for 4.5 h on day 4 (Th9) or day 5 (Th17) followed by staining for flow cytometry with the FoxP3 transcription factor buffer kit (eBioscience) as detailed below, as well as for intracellular IL-9 with anti-IL-9-PE (BioLegend 514104) and for IL-17 with anti-IL-17-PE (bioLegend 506904).

### Foxp3^+^ Treg cell induction

A single-cell suspension was prepared from the spleen and peripheral lymph nodes of Foxp3-GFP transgenic mice. CD4 + CD25-GFP-CD62Lhi cells were then isolated by MACS followed by FACS sorting (see previous sections). Sorted cells were washed twice in complete RPMI and then resuspended in complete RPMI at a concentration of 5 × 10^5^ cells per ml. CD3/CD28-coated 24 well plates (Costar) were prepared by adding 250 μl per well of CD3 and CD28 (eBioscience), both at 2 μg/ml in PBS, incubating for 2 h at 37 °C and then washing three times in PBS. In total 5 × 10^5^ cells were then added to each well in 1 ml of complete RPMI. Each well was made up to final volume of 2 ml complete RPMI, containing variable concentrations of treatment conditions (e.g., TGF-β) and IL-2 (produced in-house) at a final concentration of 100 U/ml. Cells were removed after 96 h for flow cytometric analysis.

### Transcription factor staining

For analysis of transcription factors, cells were resuspended in 400 μl fixation/permeabilisation buffer (eBioscience) and incubated at 4 °C for between 1 and 18 h. Following incubation, cells were resuspended and washed twice in 1 ml permeabilisation buffer (eBioscience). In total 50 μl of antibody or isotype control (diluted in permeabilisation buffer) was added to each sample. Cells were resuspended by gentle vortex and incubated at room temperature for 30 min. Finally, cells were washed in 2 ml of FACS buffer and resuspended in 200 μl FACS buffer for acquisition.

### Phosflow staining

MACS-purified CD4 + T cells were cultured in 100 μl in FACs tubes (BD Bioscience) at a concentration of 2–5 × 10^6^ cells/ml in serum free DMEM, supplemented with 2 mM L-gluatmine and 100 U/ml Pen/Strep (Gibco), overnight at 37 °C with 5% CO_2_. Cells were stimulated by the addition of hp-TGM (20 ng/ml), hTGF-β3 (20 ng/ml) or DMEM for 30 min or 16 h before fixation. Samples were fixed and stained using phosflow methods recommended by BD Bioscience. In brief, samples were fixed by the addition of 2 ml pre-warmed 1× Phosflow Lyse/Fix buffer (BD Bioscience) and mixed by inverting, followed by several washing steps in FACs buffer. The samples were permabilised by the addition of 1 ml of pre-chilled (0–4 °C) Phosflow Perm Buffer III (BD Bioscience) for 30 min on ice, followed by several washing steps in FACs buffer. Phosflow antibodies and isotype controls were added in 100 μl volume of FACs buffer as per manufactures instructions (BD Bioscience) and left at room temperature for 1 h in the dark. The samples were washed and resuspended in FACs buffer followed by analysis on a BD Celesta flow cytometer (BD Bioscience), using the following antibodies: anti-pAKT-PE (BD#560378), anti-p38-PE (BD #612585), or pSMAD2/3- PE (BD#562586).

### Treg suppression assays

Tregs induced as above were washed in MACS buffer and the CD4^+^CD25^+^GFP^+^ Treg population was isolated by FACS sorting. Responder cells (CD4^+^CD25^–^GFP^–^CD62L^hi^) were also isolated from a fresh Foxp3-GFP transgenic mouse. 10^4^ responder cells were added to each well of a 96 well round-bottomed plate together with 10^5^ irradiated APCs, 2 μg/ml soluble CD3 stimulation and a variable concentration of Treg. Proliferation was assessed after 72 h by thymidine incorporation.

### Continuous infusion via osmotic minipump

Alzet minipumps (Charles River UK) of 100 µl capacity were selected according to the duration of infusion required for individual experiments (model 1007D—7 days; model – 1002–14 days; model 1004–28 days). Minipumps were filled with the substance for infusion (HES, *Hp-*TGM or PBS control) and primed overnight by incubation in PBS at 37 °C.

Under general anaesthesia, abdominal fur was removed by shaving and the skin was prepared with chlorhexidine solution. The peritoneal cavity was accessed through an upper midline incision and the minipump was placed in the right paracolic gutter. Closure was in two layers with 5–0 undyed Vicryl® (Ethicon UK).

### Skin transplantation

Full-thickness skin transplantation was performed using a modified technique of that originally described by Billingham et al.^66^. Tail skin from donor mice was prepared immediately post-mortem, making a circumferential incision around the base of the tail and then extending the incision distally along the ventral midline. The tail skin was then stripped, placed into cold PBS and fashioned into three 1 × 1 cm^2^.

Recipient animals were placed under general anesthesia prior to shaving the right flank and preparing skin with chlorhexidine solution. The graft bed was prepared by dissecting skin from the right flank, taking care to preserve underlying subcutaneous adipose tissue (for microvascular blood supplementary to the graft). Optimally, the skin defect created was slightly larger than the size of the graft (1 mm at each edge), so that the graft remained taught and the risk of seroma formation was minimised. Following placement of the graft onto the graft bed, it was secured in place with methylated flexible collodion (William Ransom & Son Ltd.), applied sparingly along the wound edges. The grafts were covered with an iodine-impregnated non-adherent dressing (Inadine®, Johnson and Johnson Medical) and then secured in place with tape. Dressings were removed seven days after skin grafting under a brief general anaesthetic; any animals that managed to remove wound dressings before day 7 were excluded (prospectively) from the experiment as technical failures. Allografts were monitored on a daily basis following the removal of dressings and rejection was defined as more than 90% necrosis by surface area, or when the graft had completely left the recipient. Assessment of graft necrosis was performed in a blinded fashion – after surgery, graft recipients were placed into numbered cages; grafts were monitored according to cage number and were then matched to experimental groups for analysis at the end of each experiment.

Separately, skin grafts were harvested 7 days after transplantation and specimens were fixed in 10% buffered formalin solution overnight, then stored in 100% ethanol. Specimens were embedded in paraffin and then cut in 4 μm transverse sections. Haematoxylin and eosin (H&E) staining was then performed under automated protocol with a Gemini varistainer (Thermo Scientific), according to the manufacturer’s instructions. Histological scoring of rejection was performed in a blinded fashion by a consultant histopathologist. Scoring was performed on three histological sections of each skin graft according to features of vasculitis, folliculitis, dermal inflammation and epidermal degeneration, as described by Zdichavsky et al.^33^ Images were captured using a Leica DFC290 compound microscope and Leica Application Suite software.

### Primary fibroblast cell culture

All human samples were obtained with informed signed consent and with research ethics committee approval (10/H0504/9, 10/ H0720/12 and 12/EM/0058). Primary human lung fibroblast cell lines were generated by explant culture. Briefly, 1 mm^3^ explants were dissected from normal human lung tissue were cultured in Dulbecco’s modified eagle’s medium (DMEM) containing 20% FCS (v/v), penicillin (100 U/ml), streptomycin (100 μg/ml), and 2.5 μg/ml amphotericin B. A near confluent monolayer of fibroblasts was obtained after 3 to 4 weeks and passaged. Fibroblast cell line purity was confirmed by immunohistochemical characterization using antibodies to cytokeratin, von Willebrand factor, and desmin to rule out contamination by epithelial, mesothelial, endothelial, or smooth muscle cells. Experiments were conducted on cells between passages 3 and 8.

### In vitro fibrosis assay

Collagen biosynthesis and myofibroblast differentiation in 96 well format was measured by a high-content imaging based on a molecular crowding assay modified from a previously described method^67^. Briefly, confluent human lung fibroblasts were cultured in DMEM containing 0.4% FCS and ascorbic acid (100 μM), in the presence of mixed Ficoll 70 and Ficoll 400 (Sigma Aldrich) as molecular crowding agents. Cells were stimulated with serial molar equivalent concentrations of active TGF-β1 (R&D Systems) or *Hp*-TGM and incubated for 48 h. Cells were fixed and stained with antibody specific for human collagen 1 (Sigma Aldrich) or αSMA (Dako), fluorescent secondary antibody (Alex Fluo488) and nuclei counterstained with DAPI for per cell normalisation. Fluorescent signal was quantified on the INCELL 6000 high content system. Mean fluorescent intensity per well was calculated with 4 reads per well. Data were expressed as the mean ± SEM intensity of 4 technical replicates. EC50 values were calculated using four-parameter non-linear regression.

### Statistical analyses

All statistical analyses were performed using Prism 6.0 (Graphpad Software Inc.). For comparisons of two groups, Student’s two-tailed *t*-test was used, assuming unequal variance. When three or more groups were analysed, a one-way ANOVA test was used with Tukey’s multiple comparison test. Graft survival curves were compared by Kaplan–Meier analysis; the statistical significance of difference in survival between experimental groups was determined by a log rank chi-square test. *P* values of < 0.05 were considered to be significant; the following symbols were used to indicate significance levels: denoting *p < 0.05, denoting **p < 0.01, denoting ***p < 0.001 and denoting ****p < 0.0001. Sample sizes were chosen empirically on the basis of the laboratory’s previous experience in the calculation of experimental variability (sample sizes for each experiment were not pre-determined by individual power calculations).

### Data availability

Sequence data that support the findings of this study have been deposited in NCBI with the primary accession code MG099712. All other data are available from the corresponding author upon request.

## Electronic supplementary material

Supplementary Information

Peer Review File
