# Supplementary material for: A structurally distinct TGF-β mimic from an intestinal helminth parasite potently induces regulatory T cells
Source: Nat Commun. 2017 Nov 23;8:1741. doi: 10.1038/s41467-017-01886-6 (PMC5701006; doi:10.1038/s41467-017-01886-6)
Supplement: Supplementary file 1 — Supplementary Information [file 41467_2017_1886_MOESM1_ESM.pdf]

Suppl Fig 1

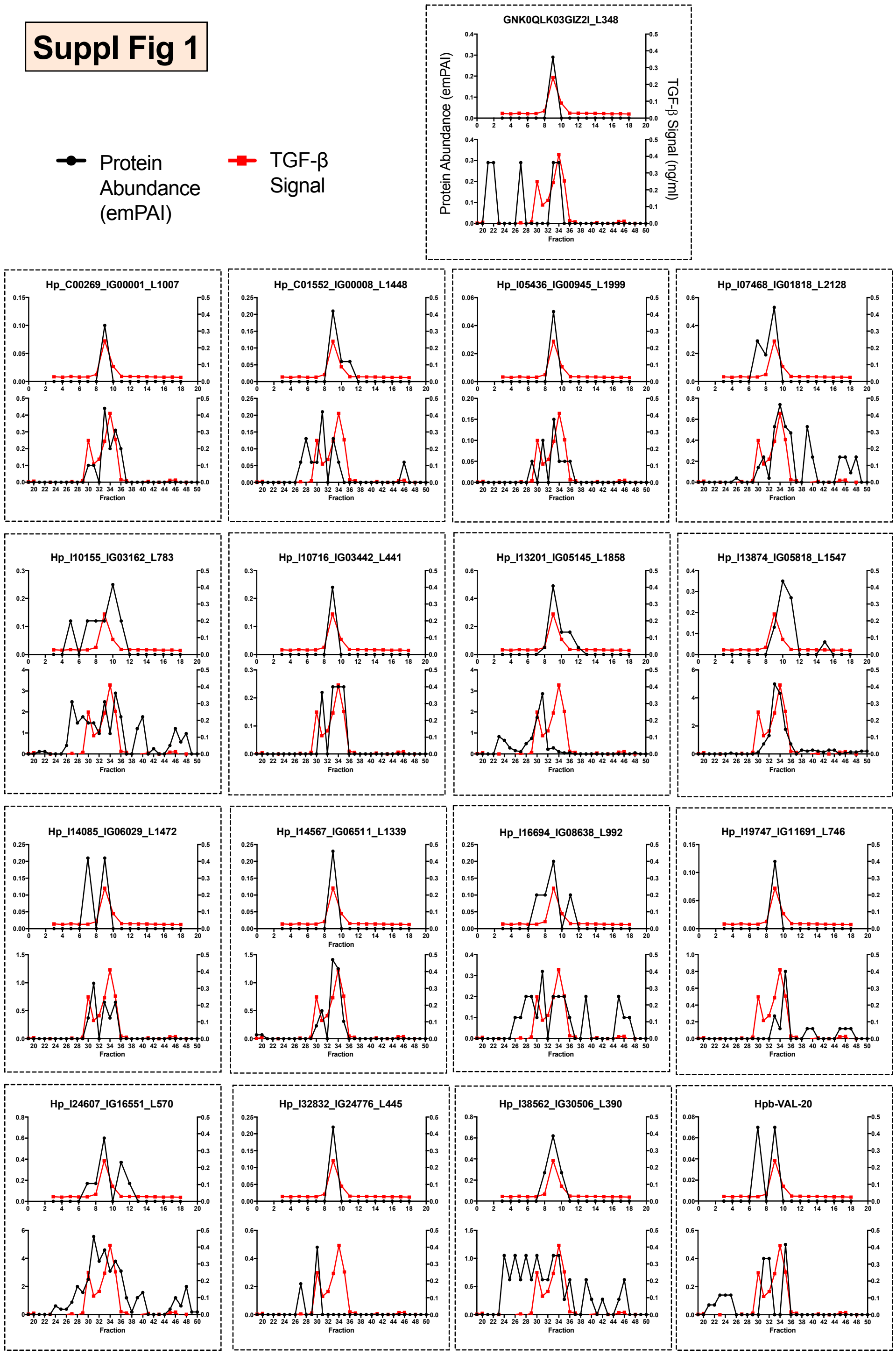

Supplementary Figure 1

Mass spectrometric profiles of candidate proteins

Protein abundance profiles for all candidate proteins found in both gel-filtration (upper panels) and ion exchange (lower panels) fractions with peak TGF-β activity, except for Hp_I03161_IG00349_L1408 shown in **Fig. 1 C**. Hp_I03161_IG00349_L1408 was the only candidate found to be active in the bioassay.

Suppl Fig 2

```
        10         20         30         40         50         60         70         80
MLLTVVIGLL EVAATDDSGC MPFSDEAATY KYVAKGPKNI EIPAQIDNSG MYPDYTHVKR FCKGLHGEDT TGWFVGICLA

        90        100        110        120        130        140        150        160
SQWYYYEGVQ ECDDRRCSPL PTNDTVSFEY LKATVNPGII FNITVHPDAS GKYPELTYIK RICKNFPTDS NVQGHIIGMC

       170        180        190        200        210        220        230        240
YNAEWQFSST PTCPASGCPP LPDDGIVFYE YYGYAGDRHT VGPVVTKDSS GNYPSPTHAR RRCRALSQEA DPGEFVAICY

       250        260        270        280        290        300        310        320
KSGTTGESHW EYYKNIGKCP DPRCKPLEAN ESVHYEYFTM TNETDKKKGP PAKVGKSGKY PEHTCVKKVC SKWPYTCSTG

       330        340        350        360        370        380        390        400
GPIFGECIGA TWNFTALMEC INARGCSSDD LFDKLGFEKV IVRKGEGSDS YKDDFARFYA TGSKVIAECG GKTVRLECSN

       410        420
GEWHEPGTKT VHRCTKDGIR TL
```

Supplementary Figure 2

Deduced amino acid sequence of Hp-TGM (H. polygyrus TGF-β Mimic).

Predicted signal peptide (aa 1-18) indicated in red; cysteine residues in yellow; 5 potential N-glycosylation sites (NxS/T) are shown in green. Peptides identified by mass spectrometry (263-243) are underlined.

Suppl Fig 3

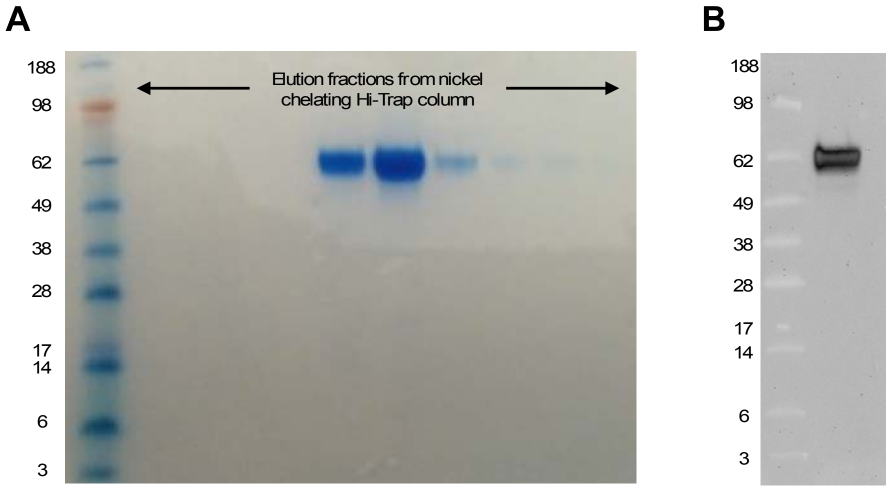

Supplementary Figure 3
Expression of recombinant *Hp*-TGM.
A. Secreted purified recombinant *Hp*-TGM from transfected HEK293T cells analysed by SDS-PAGE electrophoresis and Coomassie Blue staining.
B. Western blot analysis with anti-penta-His antibody

Suppl Fig 4

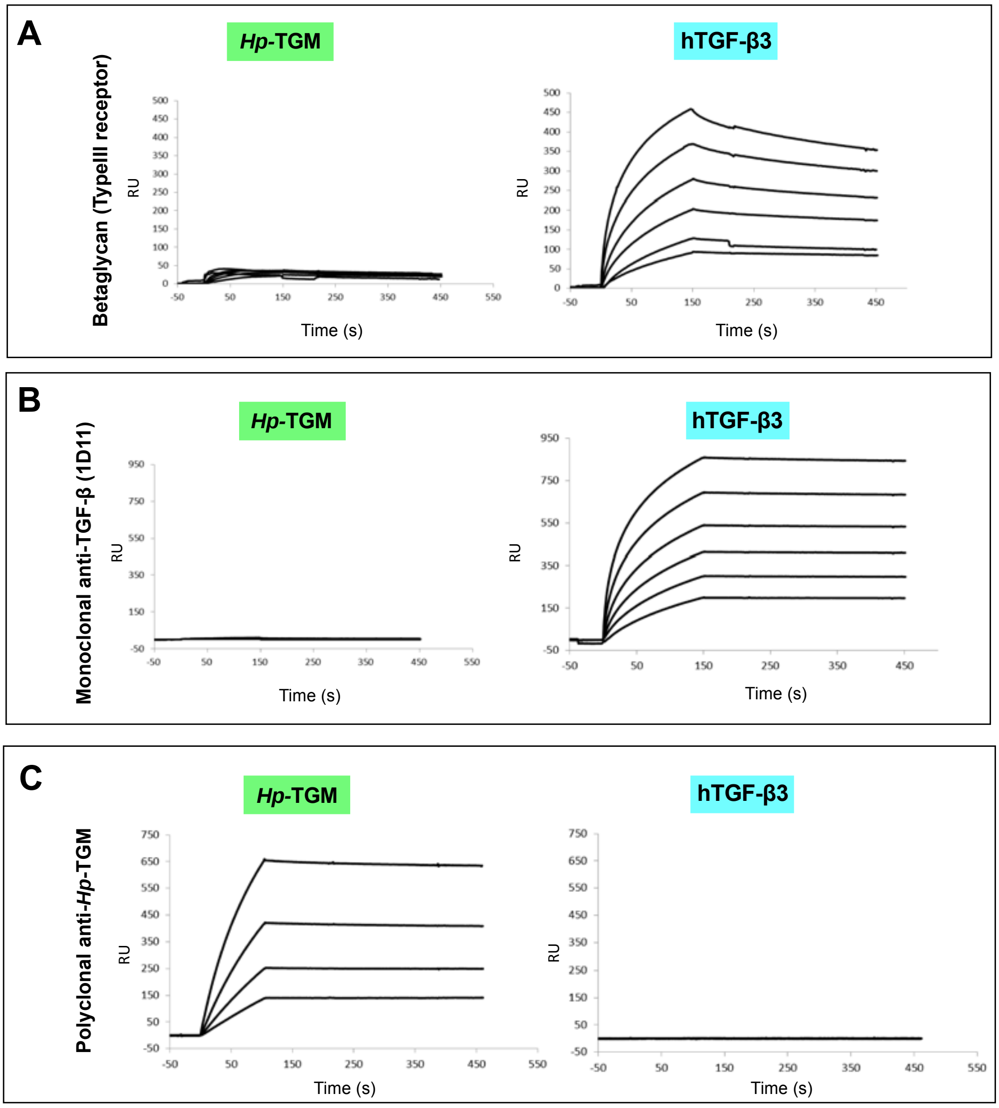

Supplementary Figure 4
Surface plasmon resonance analysis for r-betaglycan and antibody binding to immobilized *Hp*-TGM and hTGF-β3.

A. r-betaglycan binding to immobilized *Hp*-TGM (left) and hTGF-β3 (right).

B. Monoclonal antibody 1D11 specific for mammalian TGF-β binding to immobilized *Hp*-TGM (left) and hTGF-β3 (right).

C. Polyclonal rat IgG raised against *Hp*-TGM binding to immobilized *Hp*-TGM (left) and hTGF-β3 (right).

Suppl Fig 5

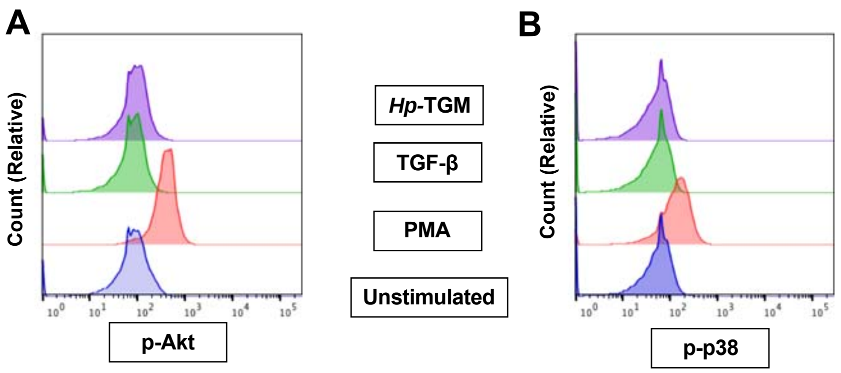

Supplementary Figure 5

***Hp*-TGM does not activate the Akt or p38 pathways**

Phospho-Flow analysis with anti-p-Akt (**A**) and p-p38 (**B**) on murine $CD4^+$ T cells stimulated for 30 mins with 20 ng/ml of *Hp*-TGM or hTGF-β1, before permeabilization and staining with specific antibody.

Suppl Fig 6

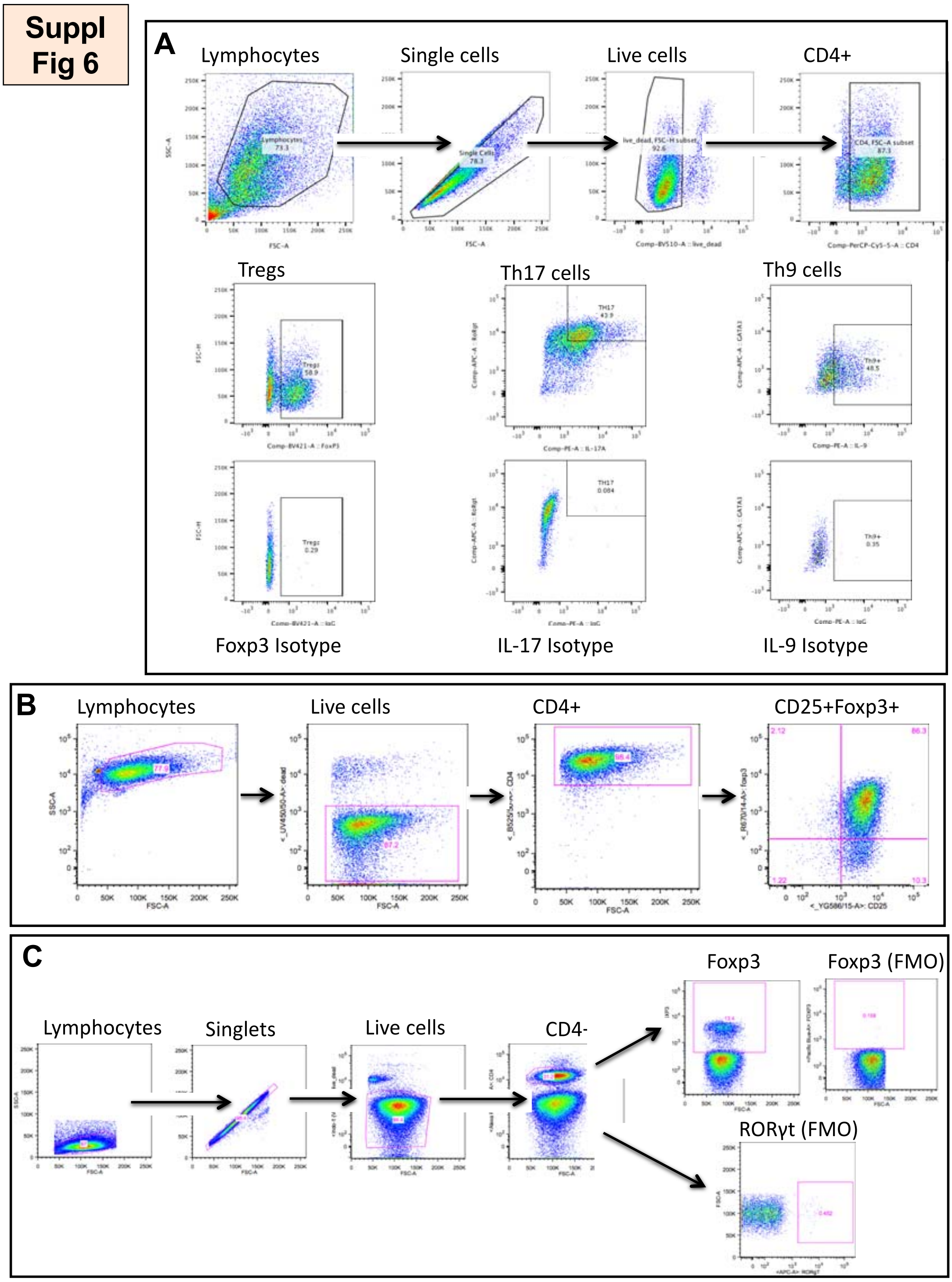

Supplementary Figure 6

Flow Cytometry gating strategies.

A. Murine splenocytes stained for CD4, Foxp3, IL-17 and IL-9 as shown in Figure 4..

B. Human peripheral blood T cells stained for CD25 and Foxp3 as shown in Figure 5.

C. Murine cells stained for Foxp3 and RORγt as shown in Figure 6.

Suppl Fig 7

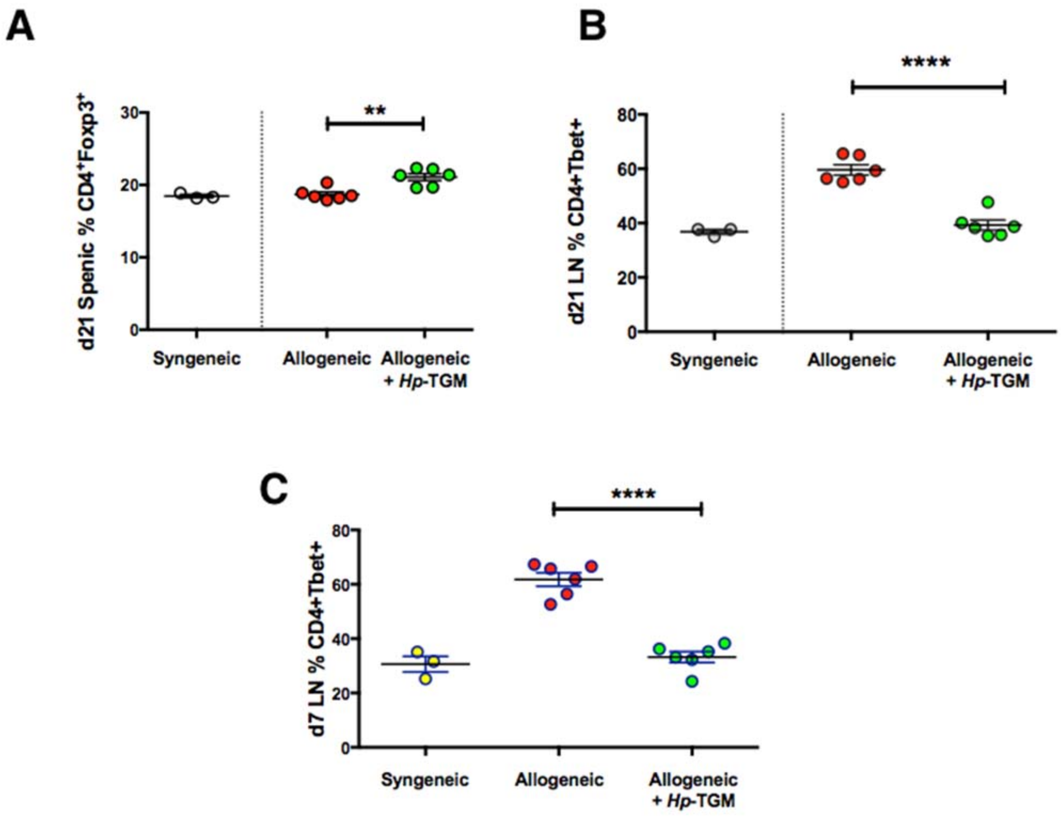

Supplementary Figure 7 : Modulation of Foxp3 and the Th1 transcription factor Tbet in $CD4^+$ T cell subsets in transplant recipients.

A. Foxp3 expression among $CD4^+$ cells in the spleen of syngeneic or fully allogeneic skin graft recipients with or without administration of *Hp*-TGM, 21 days following transplantation [syngeneic control grafts (n = 3), allograft + control protein (n = 6), allograft + TGM minipump (n = 6); comparison of groups with two-tailed, unpaired t test: p = 0.0025].

B. Tbet expression in draining lymph node of syngeneic or fully allogeneic skin graft recipients with or without administration of *Hp*-TGM, 21 days following transplantation [syngeneic control grafts (n = 3), allograft + control protein (n = 6), allograft + TGM minipump (n = 6); comparison of groups with two-tailed, unpaired t test: p < 0.0001].

C. As **B**, at 7 days post-transplantation [syngeneic control grafts (n = 3), allograft + control protein (n = 6), allograft + TGM minipump (n = 6); comparison of groups with two-tailed, unpaired t test: p < 0.0001].

Suppl Fig 8

A

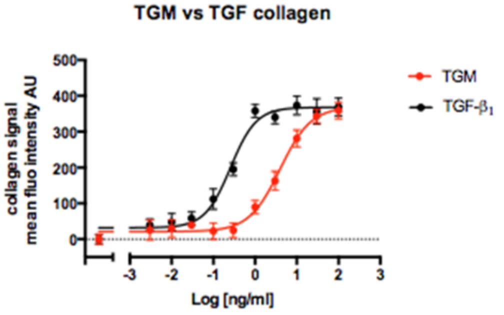

	TGM col 1	TGF col
EC50	3.960	0.2584

B

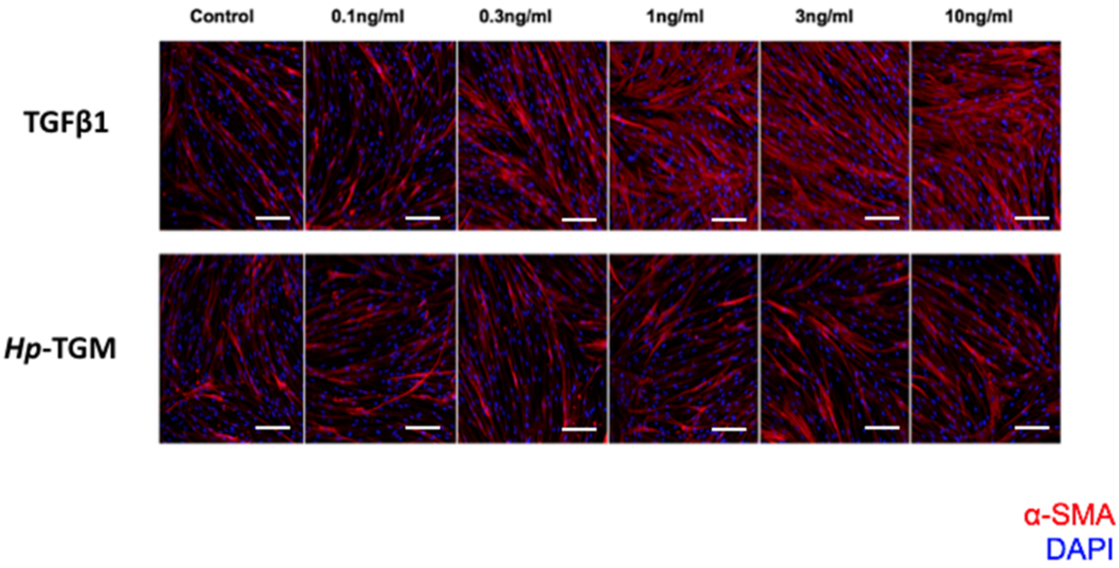

Supplementary Figure 8
Fibrogenic effects of mammalian TGF-β and *Hp*-TGM

A. Quantification of fibrogenesis assays on human lung fibroblasts *in vitro* exposed to TGFβ1 or *Hp*-TGM and assayed by immunofluorescence for collagen deposition (see **Fig. 6 G**).

B. Representative immunofluorescent images of smooth muscle actin induction by TGFβ1 or *Hp*-TGM.

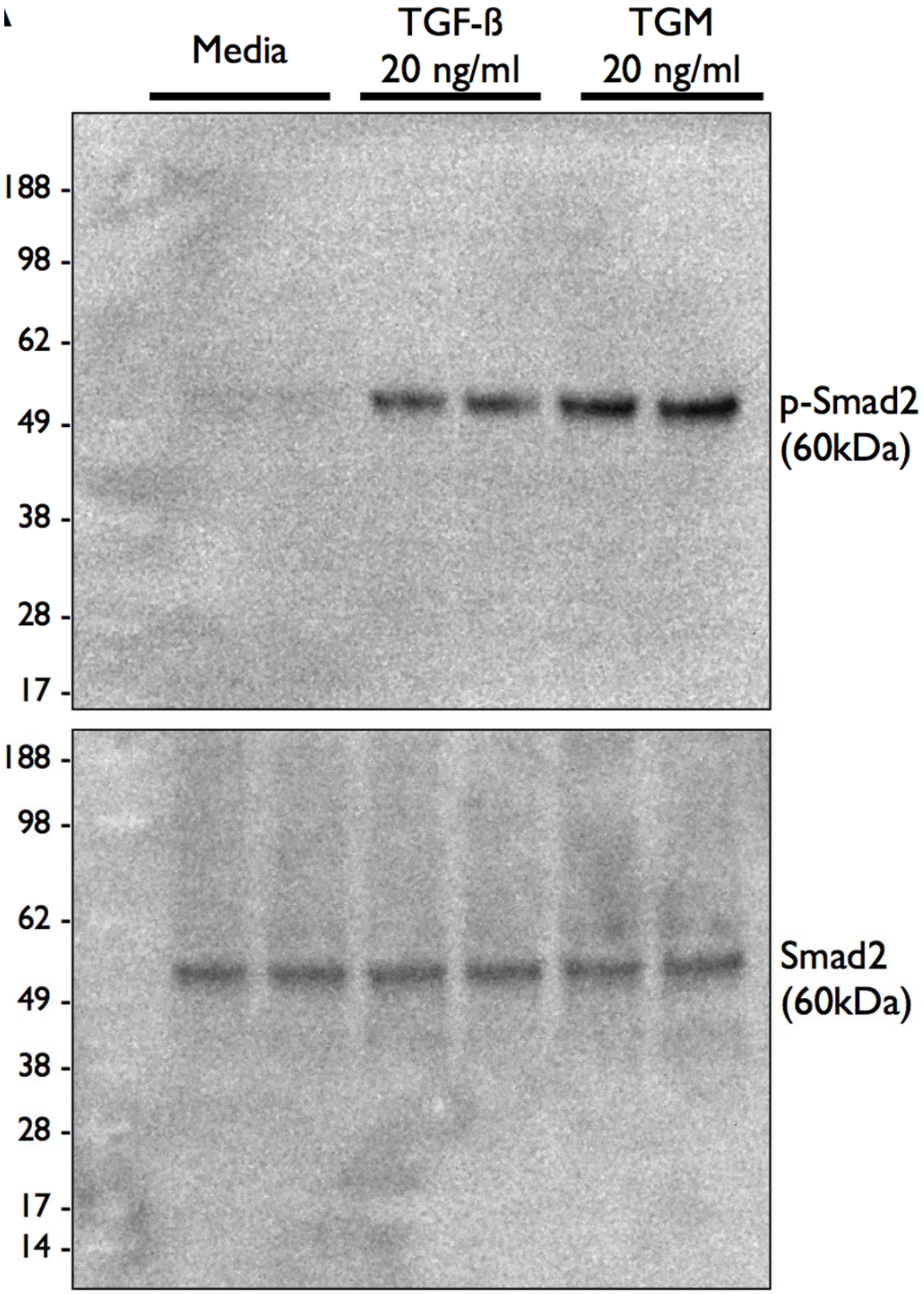

Supplementary Figure 9

Uncropped image of the Western Blot shown in Figure 3 C of cell lysates from C57BL/6 splenocytes following culture at 37°C for 18 hours probed with Smad2 and phospho-Smad2 Culture conditions in duplicate: media (DMEM+2.5% FCS), media supplemented with 20 ng/ml hTGF-β1 and media supplemented with 20 ng/ml *Hp*-TGM.

Supplementary Table 1

Abundance of 18 candidate proteins in FPLC fractions, including the active candidate including *Hp*-TGM Hp_I03161_IG00349_L1408.

Protein	Gel Filtration Fraction 9		Anion Exchange Fraction 34	
	emPAI	Rank (of 139)	emPAI	Rank (of 300)
GNK0QLK03GIZ2I_L348	0.29	138	0.29	293
Hp_C00269_IG00001_L1007	0.10	87	0.20	213
Hp_C01552_IG00008_L1448	0.21	29	0.06	202
Hp_I03161_IG00349_L1408	0.22	35	0.92	26
Hp_I05436_IG00945_L1999	0.05	130	0.05	246
Hp_I07468_IG01818_L2128	0.53	13	0.74	30
Hp_I10155_IG03162_L783	0.12	61	0.97	39
Hp_I10716_IG03442_L441	0.24	100	0.24	197
Hp_I13201_IG05145_L1858	0.49	11	0.11	140
Hp_I13874_IG05818_L1547	0.13	77	4.33	4
Hp_I14085_IG06029_L1472	0.21	47	0.37	25
Hp_I14567_IG06511_L1339	0.23	44	1.25	26
Hp_I16694_IG08638_L992	0.20	81	0.20	169
Hp_I19747_IG11691_L746	0.12	136	0.12	284
Hp_I24607_IG16551_L570	0.60	49	3.09	20
Hp_I32832_IG24776_L445	0.22	66	0	-
Hp_I38562_IG30506_L390	0.62	117	1.05	157
Hpb-VAL-20	0.07	110	0	-

Abundance is evaluated by exponentially-modified Protein Abundance Index (emPAI), and by MASCOT score which sums ion scores (1/log(P)) for each peptide identified, where P is the probabilty of matches occuring randomly.

Supplementary Table 2

Binding Constants of *Hp*-TGM and human TGF-β3 for the TGF-β receptors

**Surface**	**Analyte**	**Dissociation constant (M)**			
		**K_a (1/Ms)**	**K_d (1/s)**	**K_D (M)**	**R_{max} (RU)**
Hp-TGM	hTβR-I	$1.1e^{4}$	$3.86e^{-3}$	$3.51e^{-7}$	43
hTGF-β3	hTβR-I	n.d.	n.d.	n.d.	n.d.
Hp-TGM	hTβR-II	$1.76e^{4}$	0.0521	$2.96e^{-6}$	89
hTGF-β3	hTβR-II	$2.65e^{5}$	0.0773	$2.94e^{-7}$	305
Hp-TGM	hTβR-I*	$9.07e^{3}$	$3.15e^{-3}$	$3.48e^{-7}$	30
hTGF-β3	hTβR-I*	$4.89e^{3}$	$1.79e^{-3}$	$3.67e^{-7}$	44

* Analysis performed in presence of 2 μM of hTβR-II; n.d. Not determined due to weak binding
