# Supplementary material for: A structurally distinct TGF-β mimic from an intestinal helminth parasite potently induces regulatory T cells
Source: Nat Commun. 2017 Nov 23;8:1741. doi: 10.1038/s41467-017-01886-6 (PMC5701006; doi:10.1038/s41467-017-01886-6)
Supplement: Supplementary file 2 — Peer Review File [file 41467_2017_1886_MOESM2_ESM.pdf]

Editorial Note: This manuscript has been previously reviewed at another journal that is not operating a transparent peer review scheme. This document only contains reviewer comments and rebuttal letters for versions considered at Nature Communications.

REVIEWERS' COMMENTS:

Reviewer #2 (Remarks to the Author):

The authors have satisfactorily addressed reviewers' concerns. The manuscript is much improved and is acceptable for publication.

Reviewer #3 (Remarks to the Author):

All questions raised in my previous review have been adequately addressed.
